# Anti-inflammatory effect of pomegranate flower in lipopolysaccharide (LPS)-stimulated RAW264.7 macrophages

**DOI:** 10.1080/13880209.2017.1357737

**Published:** 2017-08-23

**Authors:** Jianjun Xu, Yongxin Zhao, Haji Akber Aisa

**Affiliations:** a Key Laboratory of Chemistry of Plant Resources in Arid Regions, Xinjiang Technical Institute of Physics and Chemistry, Chinese Academy of Sciences, Urumqi, China;; b University of Chinese Academy of Sciences, Beijing, China;; c State Key Laboratory Basis of Xinjiang Indigenous Medicinal Plants Resource Utilization, Xinjiang Technical Institute of Physics and Chemistry, Chinese Academy of Sciences, Urumqi, China

**Keywords:** Punica granatum, anti-inflammation, NF-κB, MAPK

## Abstract

**Context:***Punica granatum* L (Punicaceae) flower is an important diabetes treatment in oriental herbal medicine.

**Objective:** This study investigates the inflammation effects of pomegranate flower (PFE) ethanol extract in LPS-induced RAW264.7 cells.

**Materials and methods:** PFE (10, 25, 50, 100 μg/mL) was applied to 1 μg/mL LPS-induced RAW 264.7 macrophages *in vitro.* Levels of nitric oxide (NO), prostaglandin E_2_ (PGE_2_) and pro-inflammatory cytokines interleukin (IL)-1β (IL-1β), interleukin (IL)-6 (IL-6) and tumor necrosis factor (TNF-α) in the supernatant fraction were determined using enzyme-linked immunosorbent assay (ELISA). Expression of cyclooxygenase-2 (COX-2) and inducible nitric oxide synthase (iNOS), phosphorylation of mitogen-activated protein kinase (MAPK) subgroups extracellular signal-regulated kinase (ERK), c-Jun N-terminal kinase (JNK) and P38, as well as nuclear factor-κB (NF-κB) activation in extracts were detected via Western blot.

**Results:** 10–100 μg/mL PFE decreased the production of NO (IC_50_ value = 31.8 μg/mL), PGE_2_ (IC_50_ value = 54.5 μg/mL), IL-6 (IC_50_ value = 48.7 μg/mL), IL-1β (IC_50_ value = 71.3 μg/mL) and TNF-α (IC_50_ value = 62.5 μg/mL) in LPS-stimulated RAW 264.7 cells significantly. A mechanism-based study showed that phosphorylation of ERK1/2, p38, JNK and translocation of the NF-B p65 subunit into nuclei were inhibited by the PFE treatment.

**Discussion and conclusion:** These results show that PFE produced potential anti-inflammatory effect through modulating the synthesis of several mediators and cytokines involved in the inflammatory process.

## Introduction

*Punica granatum* L. (Punicaceae), commonly known as pomegranate, is commercially cultivated for its edible fruit in the drier regions of Southeast Asia, the Mediterranean region, and the United States (Yuan et al. 2012). According to recent reports, pomegranate is a polyphenol-rich fruit, and showed potential as an anti-inflammatory and antioxidative medicine in several experimental models (Shukla et al. 2008). The various extracts/constituents of different parts of this plant possess a number of biological activities such as antitumor, antibacterial, antifungal, and antiulcer (Celik et al. 2009). The phytochemical and pharmacological actions of all *Punica granatum* components suggest a wide range of clinical applications where inflammation is believed to play an essential etiologic role (Lansky and Newman 2007). The pomegranate flower extract also contains a large amount of polyphenols and possesses potent antioxidant and hepatoprotective property (Kaur et al. 2006). The Unani and Ayurvedic medicinal systems use the flowers of this plant as a remedy for diabetes (Huang et al. 2005). The effect and action mechanism of a methanol extract from pomegranate flowers on hyperglycemia *in vivo* and *in vitro* were investigated, and the studies indicated that pomegranate flowers improve postprandial hyperglycemia in type 2 diabetes by inhibiting glucosidase activity (Li et al. 2005). In a previous study, preliminary studies reported the anti-inflammatory effect of pomegranate flower extract (Rahima et al. 2009); however, the functional components and underlying mechanism remained uncertain. Inflammation is a common pathophysiology of many different diseases and the most primitive protective response to a variety of stimuli (Shi et al. 2009). Macrophages play critical roles in immune reactions, allergy, inflammation and they protect the body from external intruders through phagocytosis (Shi et al. 2009). During this process, macrophages produce many kinds of inflammatory mediators such as IL-1β, TNF-α, NO, and prostaglandins (Shao et al. 2013). LPS, a component of the Gram-negative bacteria cell wall, has been often used in inflammatory response because it can activate macrophages (Poltorak et al. 1998). Activated macrophages transcriptionally express iNOS, which catalyzes the oxidative deamination of l-arginine to produce NO. Excessive generation of NO by iNOS can trigger deleterious consequences such as septic shock and inflammatory diseases (Jung et al. 2014). Prostaglandins (PGs) also functions as mediators of the inflammatory response to induce pain, fever and other symptoms (Jin et al. 2010). LPS-stimulated macrophages activate several intracellular signaling pathways, including NF-κB and MAPK pathways (Guha & Mackman 2001). The present study examined the potential for PFE to reduce inflammation effects in LPS-stimulated RAW 264.7 murine macrophages *in vitro*. In order to correlate the phenolic composition with the bioactivity, high performance liquid chromatography coupled to quadruple time-of-flight with tandem mass spectrometry (HPLC/QTOF–MS/MS) was used to analyze the active components in PFE.

## Materials and methods

LPS, MTT [3-(4,5-dimethylthiazol-2-yl)-2,5-diphenyl tetrazolium bromide], phenylmethylsulfonyl fluoride (PMSF) and the components of the whole cell lysis buffer for Western blot analysis and dimethylsufoxide (DMSO) were purchased from the Sigma Chemical Co. (St. Louis, MO). Dulbecco's modified Eagle's medium (DMEM) and fetal bovine serum (FBS) were purchased from Gibco BRL (Grand Island, NY). The Griess reagent kit was purchased from Beyotime Chemical Co. (Jiang Su, China). Antibodies against COX-2, iNOS, β-actin, NF-κB p65, IκBα, Lamin B, phospho-ERK1/2, ERK1/2, phospho-p38, p38, phospho-JNK/SAPK and JNK/SAPK were purchased from Cell Signaling Technology (Danvers, MA). RAW 264.7 mouse macrophage cells were purchased from Shanghai Institutes for Biological Sciences (Chinese Academy of Sciences, China). Methanol (HPLC grade) was purchased from Fisher Scientific (Fair Lawn, NJ). All other chemicals were commercial products of reagent grade.

### Preparation of PFE

The pomegranate flower was commercial cultivar from Hotan County, Xinjiang Uygur Autonomous Region, China, and identified by Prof. Guanmian Shen (Xinjiang Institute of Ecology and Geography, Chinese Academy of Sciences). The herbarium number of PEF was 00022757 and deposited in Xinjiang Institute of Ecology and Geography. The dried pomegranate flower was extracted with ethanol under reflux three times. After removal of ethanol with rotary evaporator and under vacuum conditions at 45 °C, the residual extracts were subjected to a D-101 macroporous resin glass chromatography column with a 1:4 diameter height ratio. 70% (v/v) ethanol was used as elution solvent, and the desired fractions were concentrated *in vacuo* to produce PFE.

### Conditions of HPLC/QTOF–MS/MS

The HPLC analyses were performed using an Agilent 1200 series HPLC system (Agilent Technologies, Waldbronn, Germany), equipped with a quaternary solvent delivery system. The sample was eluted at a flow rate of 0.1 mL/min in a gradient mode of A (0.1% formic acid:water) and B (methanol): 0–25 min 10–70% B, 25–40 min 70–90% B, 40–45 min, 90–10% B. The column temperature was set at 35 °C and the injection volume was 10 μL. Mass spectrometry was performed using a QSTAR Elite LC–MS/MS system from Applied Biosystems/MDS Sciex (Concord, ON, Canada) equipped with an electrospray ionization (ESI) source. The TOF mass range was set from *m/z* 80 to 1000 and the mass range for product ion scan was *m/z* 50-1500. The collision energy (CE) was set from 10 eV to 70 eV to optimize signals to obtain maximal structure information from the ions of interest.

### Cell culture and MTT assay

The cells were cultured in DMEM medium supplemented with 10% heat-inactivated FBS, 1% streptomycin/penicillin at 37 °C in a humidified atmosphere of 5% CO_2_. The cells were treated with PFE at different concentrations and then stimulated with 1 μg/mL LPS for 18 h.

### Cell viability assay

The cells were seeded in a 96-well plate and treated with various concentrations of PFE for 24 h. The cell viability was measured by an MTT assay according to our previously described method (Tursun et al. 2016).

### Measurement of NO production

RAW264.7 cells (1 × 10^5^ cells/mL) were pre-incubated for 1 h with various concentrations of PFE and stimulated with LPS (1 μg/mL) at 37 °C for 18 h in medium. NO levels were determined by measuring nitrite levels in the culture media using Griess reagent assay according to our previously described method (Tursun et al. 2016).

### Measurement of PGE_2_, IL-6, TNF-α and IL-1β

RAW264.7 cells (1 × 10^6^ cells/well) were pretreated with different concentrations of PFE for 1 h and then stimulated with LPS (1 μg/mL) for 18 h. The concentration of PGE_2_, IL-6, TNF-α and IL-1β were assayed using the ELISA kits according to the manufacturer's instructions.

#### Western blot analysis

Protein extracts were separated by SDS-PAGE and transferred to polyvinylidene difluoride (PVDF) membranes. Protein concentrations were determined using the BCA assay. Whole cell extracts, cytosolic and nuclear proteins were extracted respectively and the western blot analysis were as described previously (Tursun et al. 2016). The membranes were blocked at room temperature for 1 h with 5% nonfat dry milk, and then incubated with each primary antibody at 4 °C overnight. Blots were visualized and quantified using a ChemiDoc system with ImageLab software (Bio Rad, CA).

### Statistical analysis

All the experiments were repeated three times. Data are expressed as the means ± standard error of the mean (SEM) for the number of experiments. Statistical significance was calculated to compare treated and control groups and determined by Student's *t*-tests. A value of *p* < 0.05 and *p* < 0.01 was considered significant.

## Results and discussion

### Phenolic compound identification

**Figure 1.**
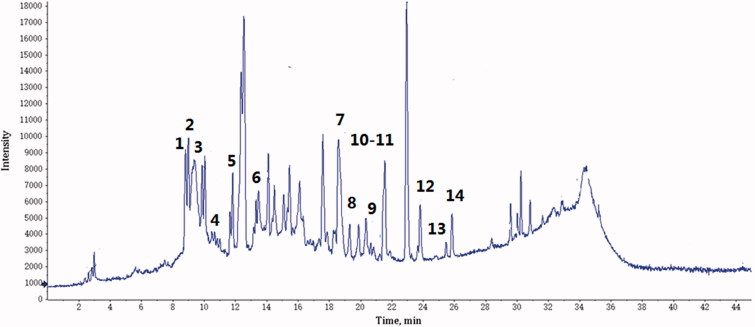
Total ion chromatograms of PFE in negative ESI mode.

Chemical compositions were separated and investigated by LC/QTOF–MS/MS in negative ESI mode at different CE values (Figure 1). The maximal structural information was obtained for the identification of components. A total of 14 compounds were identified according to accurate mass and the characteristic fragments at low and high CE. Table 1 shows the deprotonated molecular ions, retention time and characteristic fragment ions of identified compounds.

**Table 1.** Characterisation of compounds in PFE by HPLC-QTOF-MS/MS.

Peak	t_R_ (min)	[M-H]^−^	Major and important MS^2^ ions	Identification
1	8.66	633.1440	481,301, 275, 247, 203, 175, 169	Galloyl-HHDP-glucoside
2	9.01	799.1565	781, 479, 301, 273, 257	Granatin A
3	9.57	483.1295	301,275,169,125	Digalloyl-glucoside
5	11.35	635.1635	465,313,271, 169 , 125	Tri-*O*-galloyll-glucoside
6	12.14	633.1528	481, 301, 275, 247, 203, 175, 169	Galloyl-HHDP-glucoside
7	13.45	951.1904	933,463,301,275,229,167	Granatin B
8	18.44	301.0272	284, 229 ,185 145, 129	Ellagic acid
9	19.22	491.1155	453,301,275,209,177,169	Ellagic acid derivative
10	19.81	477.1152	301,275,187,151,125	Ellagic acid- rhamnoside
11	21.35	301.0651	255,179,151,121,107	quercetin
12	21.43	463.1409	301, 271 ,255, 229, 179 ,151	Quercetin-*O*-glucoside
13	23.74	285.0703	71,255,227,151	Kaempferol
14	25.41	285.6710	285,241,175,151,133	Luteolin

t_R_: retention time; HHDP: hexahydroxydiphenoyl.

### Effect of PFE on RAW 264.7 cell viability

**Figure 2.**
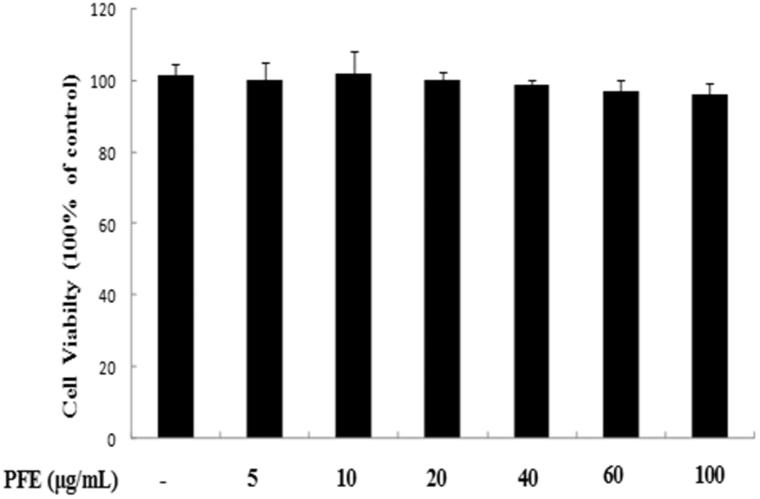
Cytotoxicity of PFE in RAW 264.7 cells. Cells were treated with different concentrations of PFE for 24 h, and viability was assayed by the MTT assay. Data represent mean values of triple determinations ± SEM. PFE at 100 μg/mL was not cytotoxic.

RAW264.7 cells were initially seeded in microplates followed by different concentrations of PFE. Treating RAW 264.7 cells with PFE (0–100 μg/mL) did not affect the viability of the RAW 264.7 cells (Figure 2).

### Effect of PFE on NO, PGE_2_ production and iNOS, COX-2 expression

**Figure 3.**
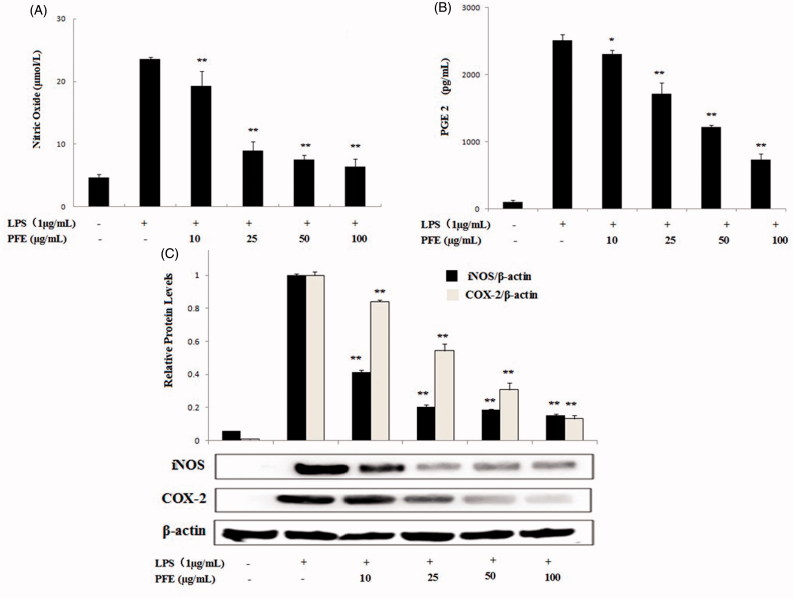
Effects of PFE on LPS-induced NO, PGE2 production and iNOS, COX-2 protein expression levels in LPS-induced RAW264.7 cells. Cells were incubated in the presence of PFE or in combination with 1 μg/mL LPS for 18 h. The culture supernatant was analyzed for NO (A), PGE_2_ (B) production. The iNOS and COX-2 (C) expression levels were determined by Western blotting. Data show mean ± SEM values of three independent experiments. *p < 0.05 and **p < 0.01 indicate significant differences from LPS-stimulation value.

The amounts of nitrite, a stable metabolite of NO, were determined via the Griess reaction. As presented in Figure 2(A,B), in unstimulated RAW 264.7 cells, NO and PGE_2_ production were almost undetectable. Upon LPS treatment, nitrite concentrations and PGE_2_ production in the medium increased markedly. The different concentrations of PFE inhibited NO and PGE_2_ production in LPS-stimulated RAW264.7 cells. We then tested the effect of PFE on iNOS and COX-2 expressions. Western blot analysis demonstrated that unstimulated RAW264.7 cells did not express iNOS and COX-2 proteins, but LPS treatment induced iNOS and COX-2 expressions. Western blotting with PFE of anti-iNOS and COX-2 antibodies showed lower iNOS and COX-2 protein levels, indicating that PFE could regulate inflammatory effects through inhibiting the iNOS and COX-2 pathway (Figure 3).

### Effects on pro-inflammatory cytokines TNF-α, IL-6 and IL-1β

**Figure 4.**
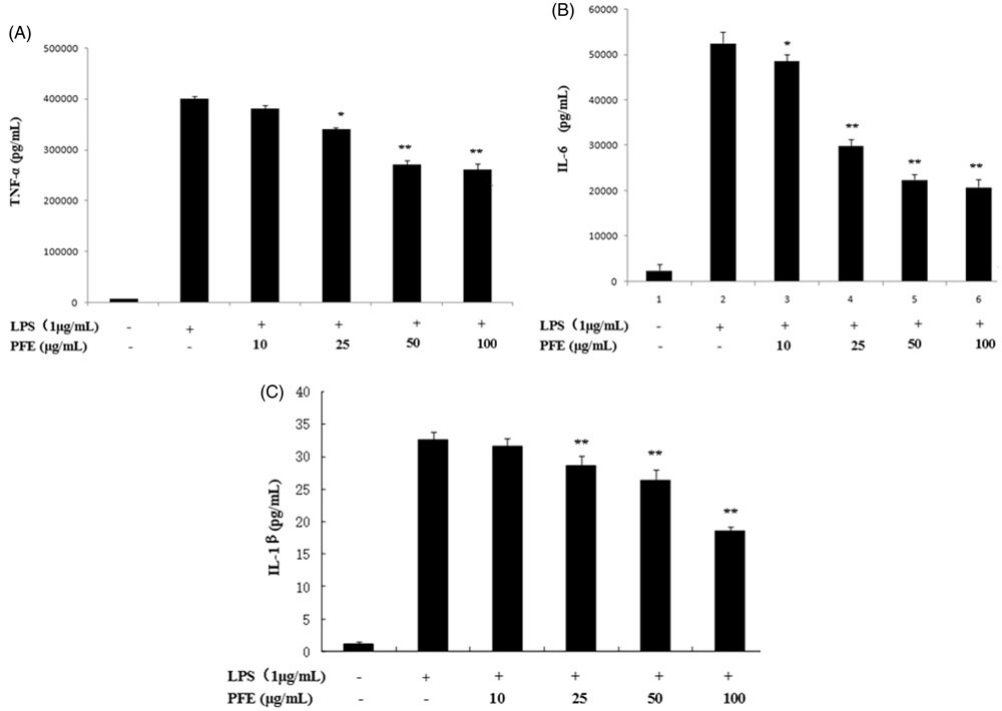
Effects of PFE on TNF-α (A), IL-6 (B) and IL-1β (C) in LPS-induced RAW264.7 cells. The cells were pretreated with the different concentrations of PFE for 1 h and then exposed to 1 μg/mL LPS for 18 h. The levels of TNF-α, IL-1β and IL-6 in the supernatant were determined by ELISA. Data show mean ± SEM values of three independent experiments. *p < 0.05 and **p < 0.01 indicate significant differences from LPS stimulation value.

In response to LPS stimulation, macrophages could release pro-inflammatory cytokines, such as TNF-α, IL-1β and IL-6. The RAW264.7 cells were treated with LPS in the presence or absence of PFE, and the levels of TNF-α, IL-1β and IL-6 were measured by ELISA. As shown in Figure 4(A–C), PFE suppressed the productions of TNF-α, IL-1β and IL-6 in LPS-induced RAW264.7 cells in a concentration-dependent manner.

### Inhibitory effect of PFE on LPS-induced signal transduction pathways

**Figure 5.**
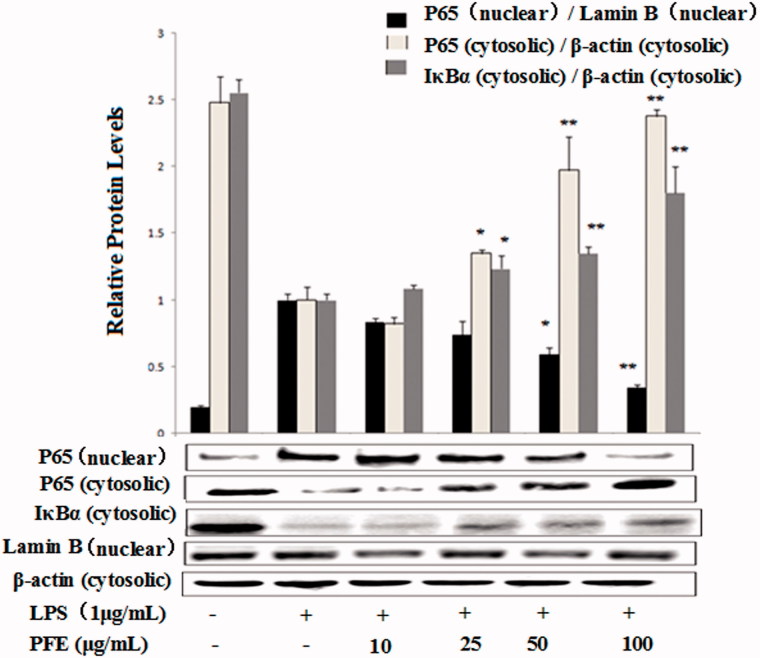
Effects of PFE on NF-κB p65 and IκBα activity in LPS-stimulated RAW 264.7 cells. The cells were pretreated with the different concentrations of PFE for 1 h and then exposed to 1 μg/mL LPS for additional 30 min. Cytoplasm and nuclear extracts proteins of cells were harvested for measurements of NF-κB p65 and IκB-α protein by Western blotting. β-Actin and Lamin B were used as the internal control. Data show mean ± SEM values of three independent experiments. *p < 0.05 and **p < 0.01 indicate significant differences from LPS-stimulation value.

The data above showed that PFE downregulated proinflammatory mediators as well as cytokines production. Under unstimulated conditions, NF-κB remains in the cytoplasm inactively bound to its inhibitor IκB and becomes active through phosphorylation and degradation of IκB and the subsequent nuclear translocation of NF-κB p65 induced by LPS (Zandi et al. 1997). As shown in Figure 5, PFE significantly inhibited LPS-induced degradation of IκB-α and nuclear translocation of NF-κB p65.

### Effect of PFE on LPS-induced protein expression of p-ERK1/2, p-JNK, and p-p38 in RAW 264.7macrophages

**Figure 6.**
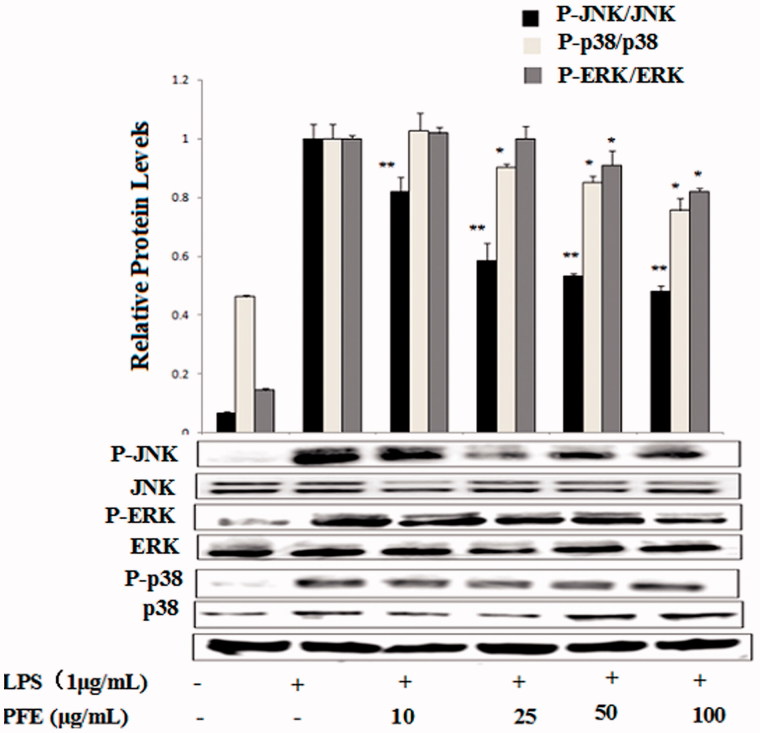
Effects of PFE on phosphorylation of MAPKs activity in LPS-stimulated RAW 264.7 cells. The cells were pretreated with the different concentrations of PFE for 1 h and then exposed to LPS for 30 min. Total cellular proteins of cells were harvested for measurements of total or phosphorylated ERK1/2, JNK, and p38 by Western blotting. Data show mean ± SEM values of three independent experiments. *p < 0.05 and **p < 0.01 indicate significant differences from LPS-stimulation value.

NF-κB is activated by phosphorylation of IκB via activation of MAPKs, which plays a key role in the signalling pathways of cell proliferation, differentiation, survival, apoptosis, and transduces various extracellular signals to the nucleus (Kim et al. 2010). In this study, we examined the effects of PFE on the phosphorylation of ERK1/2, JNK and p38. As shown in Figure 6, PFE pretreatment significantly suppressed the phosphorylation of ERK, p38 and JNK.

Pomegranate flower is an important oriental herbal medicine. The present study examined the anti-inflammatory effects of pomegranate flower using the accepted LPS-induced RAW 264.7 model. LPS stimulated macrophages trigger the secretion of cytokines and mediators, and upon treatment with LPS, RAW 264.7 cells produce induced NO and PGE_2_ as well as other inflammatory cytokines (Le et al. 2017). NO is an important inflammatory product produced by iNOS and is primarily involved in promoting inflammatory responses. Its excess production, especially in macrophages, can lead to cytotoxicity, inflammation, carcinogenicity, and autoimmune disorders (Lee et al. 2009).

PFE inhibited the pro-inflammatory mediators PGE_2_ and NO, we thereafter examined its effects on the LPS-induced production of TNF-α, IL-1β and IL-6 by ELISA. NF-κB is a transcription factor that regulates a number of genes, including iNOS, COX-2, TNF-α, IL-1β and IL-6, which are important for immunity in LPS-induced inflammation (Lee et al. 2012). In this study, we found that PFE significantly inhibited LPS-induced degradation of IkBα and the NF-κB p65 translocation from cytosol to nucleus. Activation of MAPKs is involved in LPS-induced expression of inflammatory mediators and NF-κB activation (Lee et al. 2012). It has reported that blockage of RAW 264.7 cells p38, ERK and JNK pathways causes down regulation of COX-2 expression, TNF-α and IL-1β production during inflammation (Ruberlei et al. 2017). To further investigate the mechanisms underlying the anti-inflammatory effects of PFE, phosphorylation of ERK1/2, JNK and p38 were also examined.

Phenolic compounds are plant secondary metabolites known for their antioxidative and anti-inflammatory properties (Dussossoy et al. 2011). In previous studies, quercetin has been reported to exhibit anti-inflammatory effects through regulation of nitric oxide and TNF-α production by NF-κB pathway in LPS-stimulated macrophages (Nakamura and Omura 2008). The effect of ellagic acid on inflammation also has been studied using *in vitro* and *in vivo* models (Angeles Rosillo et al. 2012; Anitha et al. 2013). Ellagic acid exerted a renal protective effect in high fat diet/low-dose streptozotocin (HFD/STZ)-induced type 2 diabetic rats by multifactorial approach (Ahad et al. 2014). The anti-inflammatory properties and underlying molecular mechanisms of luteolin in LPS RAW264.7 macrophages have been investigated. Luteolin reduced the expression of pro-inflammatory cytokines and it has potential applications as a functional food component in regulating inflammatory responses (Chen et al. 2007). In the present study, 14 chemical compositions were identified or partially characterized by LC/QTOF–MS/MS. The phenolic compounds mentioned above were identified in a previous study; therefore, it can be assumed that the anti-inflammatory effect by the PFE extracts was due to these compounds. Based on these results, pomegranate flower extracts represent potential natural sources that will be useful for the treatment of inflammatory-related diseases.

## Conclusions

The current main task of our study is to investigate the mechanisms underlying the anti-inflammatory activity of PFE in LPS-induced RAW 264.7 cells. Our findings suggest that PFE is able to inhibit the production of NO, PGE_2_, and pro-inflammatory cytokines (TNF-α, IL-6, IL-1β), as well as the protein expression of iNOS and COX2 in LPS-stimulated RAW264.7 macrophages. Moreover, PFE treatment significantly inhibited LPS-induced NF-κB activation through blocking nuclear translocation of NF-κB and IκBα degradation and PFE treatment also inhibited the phosphorylation of MAPKs. Therefore, we suggest that PFE should be considered as candidate potential anti-inflammatory agents for the treatment of inflammation-related diseases.
